# Hysteroscopic‎ polypectomy with ‎endometrial resection preventing the recurrence of endometrial polyps: A single-blinded randomized clinical ‎trial

**DOI:** 10.22088/cjim.13.2.393

**Published:** 2022

**Authors:** Mansoureh Vahdat, Ashraf Sadat Mousavi, Mania Kaveh, Kambiz Sadegi, Hoda Abdolahi

**Affiliations:** 1 1.Endometriosis Research Center, Iran University of Medical Science, Tehran, Iran; 2 Endometriosis and Gynecological Disorder Research Center, Iran University of Medical ‎Science, Tehran, Iran; 3 Department of Obstetrics and Gynecology, Zabol University of Medical Science, Zabol, Iran‎; 4 Pain Research Center, Iran University of Medical Science, Tehran, Iran; 5 Zabol University of Medical science, Anesthesiology Department, Zabol, Iran; 6 Department of Obstetrics & Gynecology, School of Medicine, Rasool Akram Teaching ‎Hospital, Iran University of Medical Sciences, Tehran, Iran

**Keywords:** Endometrium, Polyp, Recurrence, Hysteroscopy, Endometrial Ablation Techniques.

## Abstract

**Background:**

Recurrence of endometrial polyp following the hysteroscopic polypectomy is a significant concern for both the patients and physicians. This study aimed to evaluate the efficacy of combining hysteroscopic polypectomy with endometrial resection in reducing the rate of recurrence in women over 40 years old.

**Methods:**

In a single-blinded clinical trial, 94 women with endometrial polyps who were unwilling to future pregnancy were identified and randomly allocated to the intervention (hysteroscopic‎ polypectomy + endometrial resection) ‎and control group (hysteroscopic‎ polypectomy alone) group (n=47/each). Randomization was done using a simple randomization technique‎. The primary outcome measure was the polyp recurrence. The secondary outcome measure was the number of adverse events.

**Results:**

In total, polyp recurrence occurred in two (4.3%) patients of the intervention group and nine patients (19.1%) of the control group (P=0.019). All the recurrences occurred in the premenopausal patients (P=0.012). No adverse event was observed in any patients of both groups.

**Conclusion:**

Adding endometrial resection to hysteroscopic polypectomy, especially in postmenopausal women, is a safe method that significantly reduces the risk of recurrence of the endometrial polyp.

Endometrial polyps are one of the most common etiologies of abnormal genital bleeding, particularly in postmenopausal women (1). Although the exact prevalence of this type of polyp is unknown, a prevalence rate fluctuating from 7.8- 34.9% has been reported in earlier investigations (2). The definitive diagnosis is the pathological evaluation of the biopsy sample. However, the majority of cases are asymptomatic and typically detected during generalized diagnostic procedures such as transvaginal ultrasound or hysteroscopy. Abnormal uterine bleeding is the most frequent symptom of endometrial polyps, which could be associated with pain in large polyps protruding from the external cervical os (3). About 1.0% of endometrial polyps may become hyperplastic or show the malignant transformation, and the incidence of this transformation increases with age (4). Therefore, adequate treatment of endometrial polyps is of considerable importance to reduce the chance of malignancy. For a single polyp of less than 1 cm in asymptomatic postmenopausal women without risk factors, monitoring of the lesion may be sufficient (5). 

The treatment of choice for symptomatic endometrial polyps is the removal of the polyps, and hysteroscopic polypectomy is considered the “gold standard” technique for this purpose (5). However, a recurrence rate of up to 43.6% has been reported after this approach (6, 7) and this rate is significantly higher in patients with specific risk factors (breast cancer and Tamoxifen treatment) (8). Therefore, the development of new techniques is necessary to prevent polyp recurrence after resection. Endometrial resection is a uterine lining ablation technique using a wire-loop resectoscope under hysteroscopic guidance (9). 

It is known as a safe and effective method for heavy menstrual bleeding (10). Recently, post-polypectomy endometrial resection has proven to be effective in preventing the recurrence of endometrial polyps. However, only a few studies have evaluated the effect of post-polypectomy endometrial resection as a prophylactic method for preventing recurrence of polyp (11-14). In this study, we aimed to compare the effect of post-polypectomy endometrial resection with hysteroscopic polypectomy on the recurrence rate of endometrial polyps in patients aged 40 yrs. and older.

## Methods

Ninety-four patients with endometrial polyp diagnosis who were referred to our university hospital between January and December 2018 and had indications for hysteroscopic polypectomy were included in this single-blinded randomized clinical ‎trial study. Patients were randomly assigned to two study groups (intervention and control), 47 patients each. The main symptom was abnormal uterine bleeding that was noticed in all patients. 

**Figure 1:**
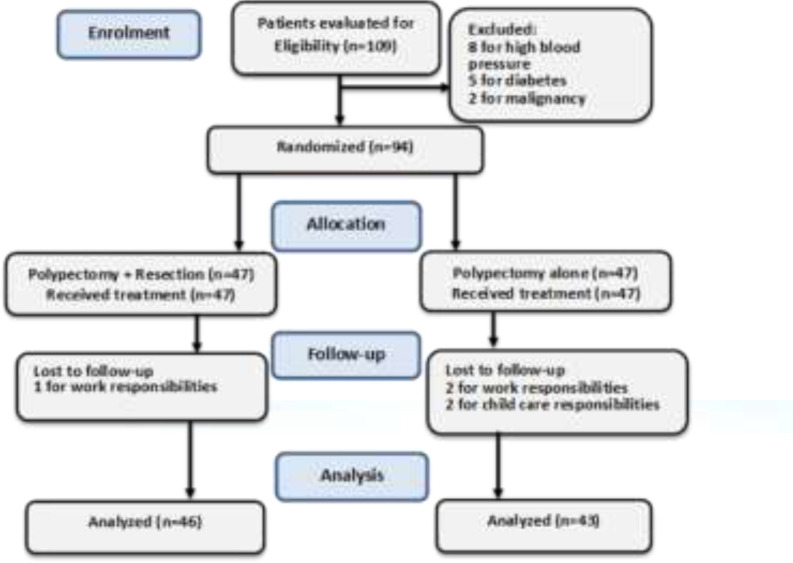
Flow diagram of the study inclusion and exclusion

**Inclusion and exclusion criteria: **The inclusion criteria were as follows: (i) patients aged 40 years and older (ii) sonographic diagnosis of endometrial polyps, (iii) not taking Tamoxifen, (iv) unwillingness to get pregnant, and (v) satisfaction to participate in the study. The exclusion criteria were as follows: (i) having high blood pressure, (ii) diabetes, and (ii) endometrial malignancy that was checked by endometrial sampling using Pipelle. The flow diagram of patients’ inclusion and exclusion is demonstrated in figure 1.

**Randomization technique: **Random assignment was done using a simple randomization technique. For this purpose, the patients were numbered consecutively. Then, a random number list was generated by the RAND function of Excel software. Subsequently, the random numbers were sorted in ascending order. The first 47 patients were then allocated to the intervention group. The remaining 47 patients were assigned to the control group. Randomization was performed by a nursing assistant who was blinded to the intervention type.

**Surgical procedure: **Cervical and endometrial malignancy was ruled out before the surgery. In the operating room, the patients were placed in a lithotomy position. The type of anesthesia depended on the patient’s characteristics. All women underwent hysteroscopic polypectomy techniques. In the control group, no further therapeutic procedure was performed. In the intervention group, hysteroscopic polypectomy was followed by endometrial resection. A 250×4 mm optical resectoscope was implicated for this purpose. All the procedure was performed by one surgeon. 

**Outcome measures: **The primary outcome measure was the recurrence of the polyp that was evaluated three and six months after the surgery, using transvaginal sonography (TVS). In case a lesion was observed in TVS, a second hysteroscopy was performed to confirm the recurrence.‎ The secondary outcome measures were the adverse effects attributed to each surgical technique.

**Sample size calculation: **The sample size was calculated based on the study of Preutthipan et al., who reported a recurrence rate of 15% for endometrial polyp following the hysteroscopic polypectomy in postmenopausal women ^15^. Accordingly, a sample size of 47 patients in each group was found to be sufficient to detect a clinically significant difference between the two study groups using a chi-squared test with a power of 80% and the significance level of 5%. 

**Ethical Considerations: **This study was conducted in accordance with The Helsinki Declaration**.** All patients signed a copy of the informed consent form before participation in the study. This single-blinded randomized clinical ‎trial study was approved by the Ethics Committee of our university under the code of IR.IUMS.REC.1396.30651. The protocol of the study was also registered at the Iranian Registry of Clinical Trials (Code: IRCT 20150909023949N7). 

**Statistical analysis: **The statistical analysis was performed using SPSS Version 16 (Chicago, Illinois, USA). Kolmogorov-Smirnov test was used to check the normality of data distribution. An independent t-test was used to compare the two study groups. A chi-square test was used for the comparison of categorical variables. Intention-to-treat analysis was done using the Last Observation Carried Forward (LOCF) imputation method. A per-protocol analysis was done with completers. A p-value < 0.05 was considered significant. 

## Results

Forty-seven patients with endometrial polyps were included in each study group. Five patients in the intervention group and six patients in the control group were postmenopausal. No significant difference was observed between the characteristics of the two study groups, including age, BMI, polyp features, etc. The baseline characteristics of the participants are demonstrated in table 1 in detail. One woman in the resection group and four women in the non-resection group were lost to follow-up. In the first three months after the surgery, endometrial polyp recurred in one patient (2.1%) of the intervention group and four patients (8.5%) of the control group.

 In the second three months after the surgery, recurrence of the endometrial polyp was observed in one other patient (2.1%) of the intervention group and five other patients (10.6%) of the control group. In total, polyp recurrence occurred in two (4.3%) patients of the intervention group and nine patients (19.1%) of the control group. This difference was statistically significant in the intention-to-treat analysis (OR=5.3, 95%CI: 1.085-26.18, P=0.025). This difference was statistically significant in the per-protocol analysis, as well (OR=5.8, 95%CI: 1.180-28.73, P=0.02). All the recurrences occurred in the premenopausal patients so that the recurrence rate was statistically higher in this group of patients both in the intention-to-treat analysis (OR=1.258, 95%CI: 1.116-1.360, P=0.018) and per-protocol analysis (OR=1.482, 95%CI: 1.122-1.661, P=0.012). A significant association was also found between the number of polyp and rate of recurrence so that the majority of recurrences (8 out of 11, 72.7%) occurred in patients with more than two polyps (Intention-to-treat analysis: OR=1.803, 95%CI: 1.071-3.151, P=0.023, per-protocol analysis: OR=1.944, 95% CI:1.205-3.563, P=0.02).

 No other significant association was found between the recurrence rate and patients' characteristics such as polyp diameter, gravidity, and fertility status. No adverse event was observed in any patients of both groups. 

**Table 1:** The characteristic features of the two study groups

**Variable**	**Intervention group** **(polypectomy+ resection)** ** (n=47)**	**Control group** **(** **polypectomy) (n=47)**	**P-value**
Age (year)*	48.1 ± 9.6	46.9 ± 8.9	0.23
BMI (kg/m^2^)*	29.2 ± 4.1	28.7 ± 5.2	0.32
Mean gravidity*	3.1 ± 1.1	2.9 ± 1	0.21
Menopausal status**			
Premenopausal	42 (89.3)	41 (87.2)	0.52
Postmenopausal	5 (10.7)	6 (12.8)	
Infertility**			
Yes	2 (4.3)	4 (8.5)	0.4
History of Hysteroscopy**			
Yes	1 (2.1)	2 (4.3)	0.61
Polyp number**			
One	33 (70.2)	32 (68.1)	0.48
Two	4 (8.5)	4 (8.5)	
>Two	10 (21.3)	11 (23.4)	
Mean polyp diameter (cm)*	1.8±1.1	1.7±0.9	0.55

* Data are presented as mean±SD. ** Data presented as number (%). A p value <0.05 is considered significant. Categorical data were compared with a chi-square test. Numerical data were compared with an independent t-test.

**Table 2:** Comparison of polyp recurrence between the two study groups

**Variable**	**Intervention group** **(polypectomy+ resection)** ** (n=47)**	**Control group** **(** **polypectomy) (n=47)**	**P-value**
Polyp recurrence after 3 months	1(2.1)	4 (8.5)	0.16
Polyp recurrence after 6 months	1 (2.1)	5 (10.6)	0.1
Total number of polyp recurrence	2 (4.2)	9 (19.1)	0.02

Data are presented as number (%). A p value <0.05 is considered significant (chi-square test).

## Discussion

In this study, we evaluated the effect of endometrial resection on reducing the rate of recurrence of endometrial polyp following hysteroscopic polypectomy. Based on the result of this study, endometrial resection significantly reduced the rate of recurrence after polypectomy so that the rate of recurrence was 4.3% in patients who underwent endometrial resection and 19.3% in those who did not. No adverse event was observed in any patients of both groups. 

Maia et al.aimed to evaluate the efficacy of polypectomy associated with endometrial resection for the treatment of endometrial polyp in 66 postmenopausal women. During a one-year follow-up of the patients, they observed no recurrence in women who completed follow-up. Moreover, no major complications were associated with this procedure. They concluded that polypectomy combined with endometrial resection is a very low-risk procedure for postmenopausal patients, which significantly reduces the rate of recurrence ^12^. Similar results were observed in the current series.

Jiménez-Lopez et al. aimed to determine the efficacy of post-polypectomy hysteroscopic endometrial resection in preventing the recurrence of endometrial polyps in 362 postmenopausal patients. The success rates in the study were 99.5%, 97%, 95.7%, and 95%, corresponding to the follow-up time point 6 months, 18 months, 42 months, and 60 months, respectively. They concluded that endometrial resection is an effective and safe method in preventing the recurrence of endometrial polyps (11). The results of the present study were in accordance with the results of the study of Jiménez-Lopez and co-authors.

The study of Giampaolino et al. (14) and Elyashiv et al. (13). Showed the efficacy of endometrial resection in the management of premalignant and malignant endometrial polyps. However, to the best of our knowledge, the efficacy of hysteroscopic polypectomy combined with endometrial resection in the management of benign polyps has not been evaluated in any other investigations. Even so, hysteroscopic polypectomy combined with endometrial ablation has been studied in many investigations. Gao et al. aimed to investigate the role of hysteroscopic polypectomy combined with ‎endometrial ablation in the management of tamoxifen-associated endometrial polyps‏. They reported a recurrence rate of 5.3% following this approach. They concluded that great symptomatic relief and low recurrence rate of this approach is encouraging and warrens its application in future workouts (16). The study of Henriquez et al. was the only controlled trial aiming at the estimation of the effectiveness of hysteroscopic polypectomy, whether combined with endometrial ablation or insertion of a levonorgestrel-releasing intrauterine device or not. According to the results of this study, 4-yr-intervention-free survival was 41.1 for patients who underwent only hysteroscopic polypectomy and 54.7% for patients who underwent a combined treatment (17). The present study revealed a higher rate of recurrence postmenopausal patients so that none of the recurrences was observed in the premenopausal group of patients. This might suggest a wider application of this procedure in postmenopausal patients. However, since the number of postmenopausal women was small in this study, further investigations are needed to achieve a consensus in this regard. In addition, the present study revealed a higher risk of recurrence in patients with multiple polyps. Such association was noticed in earlier investigations as well (8). Considering the effectiveness of post-polypectomy endometrial resection in the prevention of the recurrence of endometrial polyp, we suggest performing this technique for patients with a high risk of recurrence, such as those with multiple polyps.

Altogether, the results of the present study, in line with the results of earlier investigations, reveal that a combination of hysteroscopic polypectomy and endometrial resection could be used as a more effective approach for the prevention of recurrence of the endometrial polyp. However, this study was not without limitations. The main limitation of the study was the relatively short follow-up of the patients. Moreover, the small number of postmenopausal women could have affected the power of the statistical analysis.

In conclusions, Hysteroscopic polypectomy, combined with endometrial resection is a safe method that significantly reduces the risk of polyp recurrence when compared with hysteroscopic polypectomy alone. Therefore, we suggest performing this procedure to prevent polyp recurrence, particularly in premenopausal women and patients with higher numbers of polyps. 

## References

[1] Al Chami A, Saridogan E (2017). Endometrial polyps and subfertility. J Obstet Gynecol India.

[2] Salim S, Won H, Nesbitt-Hawes E, Campbell N, Abbott J (2011). Diagnosis and management of endometrial polyps: A critical review of the literature. J Minim Invasive Gynecol.

[3] Clark TJ, Stevenson H (2017). Endometrial polyps and abnormal uterine bleeding (aub-p): What is the relationship, how are they diagnosed and how are they treated?. Best Pract Res Clin Obstet Gynaecol.

[4] Wethington SL, Herzog TJ, Burke WM (2011). Risk and predictors of malignancy in women with endometrial polyps. Ann Surg Oncol.

[5] Nijkang NP, Anderson L, Markham R, Manconi F (2019). Endometrial polyps: Pathogenesis, sequelae and treatment. SAGE Open Med.

[6] Paradisi R, Rossi S, Scifo MC (2014). Recurrence of endometrial polyps. Gynecol Obstet Invest.

[7] Al Hilli MM, Nixon KE, Hopkins MR (2013). Long-term outcomes after intrauterine morcellation vs hysteroscopic resection of endometrial polyps. J Minim Invasive Gynecol.

[8] Yang JH, Chen CD, Chen SU, Yang YS, Chen MJ (2015). Factors influencing the recurrence potential of benign endometrial polyps after hysteroscopic polypectomy. PloS One.

[9] Lethaby A, Penninx J, Hickey M, Garry R, Marjoribanks J (2013). Endometrial resection and ablation techniques for heavy menstrual bleeding. Cochrane Database Syst Rev.

[10] Fergusson RJ, Bofill Rodriguez M, Lethaby A, Farquhar C (2019). Endometrial resection and ablation versus hysterectomy for heavy menstrual bleeding. Cochrane Database Syst Rev.

[11] Jiménez-Lopez JS, Miguel AG, Tejerizo-Garcia A, Muñoz-Gonzalez JL, Lopez-Gonzalez G (2015). Effectiveness of transcervical hysteroscopic endometrial resection based on the prevention of the recurrence of endometrial polyps in postmenopausal women. BMC Womens Health.

[12] Maia Jr H, Calmon L, Marques D (1997). Polypectomy and endometrial resection in postmenopausal patients. J Am Assoc Gynecol Laparosc.

[13] Elyashiv O, Sagiv R, Kerner R (2017). Hysterscopic resection of premalignant and malignant endometrial polyps: Is it a safe alternative to hysterectomy?. J Minim Invasive Gynecol.

[14] Giampaolino P, Sardo ADS, Mollo A (2019). Hysteroscopic endometrial focal resection followed by levonorgestrel intrauterine device insertion as a fertility-sparing treatment of atypical endometrial hyperplasia and early endometrial cancer: A retrospective study. J Minim Invasive Gynecol.

[15] Preutthipan S, Herabutya Y (2005). Hysteroscopic polypectomy in 240 premenopausal and postmenopausal women. Fertil Steril.

[16] Gao W, Zhang L, Li W (2012). Three-year follow-up results of polypectomy with endometrial ablation in the management of endometrial polyps associated with Tamoxifen in chinese women. Eur J Obstet Gynecol Reproduc Biol.

[17] Henriquez DD, van Dongen H, Wolterbeek R, Jansen FW (2007). Polypectomy in premenopausal women with abnormal uterine bleeding: Effectiveness of hysteroscopic removal. J Minim Invasive Gynecol.

